# Macrophage Capping Protein CapG Is a Putative Oncogene Involved in Migration and Invasiveness in Ovarian Carcinoma

**DOI:** 10.1155/2014/379847

**Published:** 2014-04-02

**Authors:** J. Glaser, M. H. D. Neumann, Qi Mei, B. Betz, N. Seier, I. Beyer, T. Fehm, H. Neubauer, D. Niederacher, M. C. Fleisch

**Affiliations:** ^1^Department of Nephrology, University Hospital Duesseldorf, Heinrich-Heine University, 40225 Duesseldorf, Germany; ^2^Department of Obstetrics and Gynecology, University Hospital Duesseldorf, Heinrich-Heine University, 40225 Duesseldorf, Germany; ^3^Department of Oncology, Tongji Hospital, Tongji Medical College, Huazhong University of Science and Technology, Wuhan 430030, China; ^4^Institute of Human Genetics, University Hospital Duesseldorf, Heinrich-Heine University, 40225 Duesseldorf, Germany

## Abstract

The actin binding protein CapG modulates cell motility by interacting with the cytoskeleton. CapG is associated with tumor progression in different nongynecologic tumor entities and overexpression in breast cancer cell lines correlates with a more invasive phenotype *in vitro.* Here, we report a significant CapG overexpression in 18/47 (38%) of ovarian carcinomas (OC) analyzed by qRealTime-PCR analyses. Functional analyses in OC cell lines through siRNA mediated CapG knockdown and CapG overexpression showed CapG-dependent cell migration and invasiveness. A single nucleotide polymorphism rs6886 inside the CapG gene was identified, affecting a CapG phosphorylation site and thus potentially modifying CapG function. The minor allele frequency (MAF) of SNP rs6886 (c.1004A/G) was higher and the homozygous (A/A, His335) genotype was significantly more prevalent in patients with fallopian tube carcinomas (50%) as in controls (10%). With OC being one of the most lethal cancer diseases, the detection of novel biomarkers such as CapG could reveal new diagnostic and therapeutic targets. Moreover, in-depth analyses of SNP rs6886 related to FTC and OC will contribute to a better understanding of carcinogenesis and progression of OC.

## 1. Introduction

Ovarian cancer globally represents the fifth leading cause of cancer-related death among women [1]. As no effective screening is available, it is mostly diagnosed in advanced and metastatic stages [2] and therefore it is associated with a poor overall prognosis. Despite multimodal treatment strategies including surgery, chemo-, and recently antiangiogenic therapy, the average five-year overall survival of advanced stages does not exceed 40% (American Cancer Society, 2013) and the ratio of new diagnosis and death is with 1.4 most unfavorable [3].

Ovarian carcinoma has traditionally been classified histologically into serous, mucinous, endometrioid, clear-cell, and Brenner tumors, but a new tumor progression model has just recently led to a dualistic classification based on cellular invasiveness and genetic alterations [4]. Type I tumors mostly develop from precursor lesions, are slow growing and in 65% show mutations in the* braf* and* kras* genes, which play a crucial role in the cell growth signaling cascade [5]. Their development is therefore thought to follow the adenoma-to-carcinoma sequence [6].

In contrast, type II or high-grade tumors evolve rapidly with early metastasis within the peritoneal cavity. No precancerous components allow for an early detection; the median survival averages 30 months only. In 50–80% they harbor various p53 mutations [7] and an overall genetic instability is found, while a certain sequence has not yet been identified.

Recognizing that migration and invasiveness play an important role, especially in high-grade ovarian carcinoma development, we focused our study on CapG, an actin binding protein that promotes cellular motility and has previously been associated with increased invasiveness in breast cancer [8, 9].

CapG has been identified as an oncogene in various carcinomas [10–12]. In profiling array analyses, we have formerly shown an increased CapG expression in breast and ovarian cancer [13]. It is a member of the gelsolin protein family, which comprises cytoplasmic and nuclear proteins. These, among others, are involved in the shaping of the cytoskeleton by remodeling of actin filaments. This 39 kDa protein is found in both compartments, the cytoplasm and the nucleus [14], and contributes up to 1% to the entire protein amount in macrophages. It modulates actin length by capping its plus or so-called barbed ends in a Ca^2+^- and PIP_2_-dependent manner. Like all gelsolin-related proteins, it is structured in homologue domains and lacks, in contrast to other nuclear gelsolin-like proteins, a nuclear export sequence [15] while entering the nucleus is importin *β* dependent. However, it has no canonical nuclear localization signal (NLS) [16].

Previously, the unique role of CapG, independent of gelsolin, for macrophage motility, phagocytosis, and membrane ruffling has been shown in CapG knockdown mice [17]. Further, CapG and in particular its nuclear fraction has been postulated to promote cellular invasion in collagen* in vitro* [8, 16].

In the current study, we investigate CapG expression by qRT-PCR analyses. Moreover, we establish an examinable design to determine the impact of CapG on migration and invasiveness in OC cell lines.

Finally, by analyzing a single nucleotide polymorphism localized in exon 10 of the CapG gene, we suggest a novel link between fallopian tube and ovarian carcinomas.

## 2. Material and Methods

### 2.1. Patients and Tissue Samples

After receiving IRB approval, a total of 47 OC tissue samples and 21 normal adjacent tissues were obtained from a consecutive series of patients treated with surgery for ovarian cancer between 1994 and 2006 at the Department of Gynecology and Obstetrics, Heinrich-Heine-University Hospital, Duesseldorf, Germany. The median age of 47 patients with OC was 55 yrs (range: 18 to 83 yrs) and 54 yrs (24 to 71 yrs) among cases where normal tissue was obtained. FIGO classification was documented in 41/47 cases with 76% of these assigned to stages III and IV, while the majority displayed dedifferentiated tissues (grading ≥ 2). Samples were preserved in liquid nitrogen and stained with hematoxylin and eosin for tumor verification.

For SNP analyses, DNA samples (isolated from EDTA blood samples) from independent sets of 263 OC patients, 12 FTC patients, and 107 healthy controls from women aged older than 50 years (range: 52–74 yrs, mean age: 66 yrs) were collected. The age of OC patients ranged from 16 to 90 years with a mean of 61 yrs. Among fallopian tube carcinoma patients, the mean age was 70 yrs (range 44–78; *n* = 12). Samples from OC and FTC cases were obtained from patients treated with surgery and/or chemotherapy between 1997 and 2006 in our clinic. Control samples were collected from healthy women without any oncologic diseases participating in an endocrine-related study at the Department of Gynecology and Obstetrics, Heinrich-Heine-University Hospital, Duesseldorf, Germany.

### 2.2. Quantitative RealTime-PCR

Analyses were performed on 7500 Fast-RealTime Systems (Applied Biosystems, USA). RNA was isolated from samples using the RNeasy Mini Kit (Qiagen, Germany) according to the manufacturer's protocol. 1 *μ*g RNA was processed for cDNA synthesis (Omniscript RT Kit, Qiagen, Germany) and specific primers (CapG Forward 5′-cga aca ctc agg tgg aga tt-3′; Reverse 5′-tcc agt cct tga aaa att gc-3′; GAPDH Forward 5′-cct gca cca act gct tag-3′; Reverse 5′-tgg cag tga tgg cat gga gtg-3′) were used in qRT-PCR. Relative gene expression was calculated by the ΔΔCT method using GAPDH as housekeeping gene.

### 2.3. Cell Culture

Ovarian cancer cell lines SK-OV-3 (ATCC number HTB-77) and Hey (ATCC number CLU-302) were maintained in RPMI1640 medium (GIBCO, Invitrogen, Life Technologies, USA) containing 2 mg/mL D-glucose, 1% L-glutamine (GIBCO), 10% fetal calf serum (FCS Gold Mycoplex, PAA, Austria), and 0.1% Gentamicin (GIBCO). Cultivation was carried out standardized (37°C; 5% CO_2_; 95% humidity; incubator Heraeus, Germany).

### 2.4. Retroviral Expression of CapG

For stable CapG overexpression in Hey cells, a PCR-amplified fragment of human CapG (1047 bp) supplemented with restriction sites was digested following the manufacturer's instructions (New England Biolabs, UK) and cloned into the retroviral expression vector S11IN (kindly provided by H. Hanenberg) using bacteria strains DH5*α*, SURE, and XL-1 BLUE and the Endofree Plasmid Maxi Kit (Qiagen, Hilden, Germany) for plasmid extraction. Neomycin resistance was integrated for selection.

Retrovirus producer line EcoR-Phoenix was transfected with either expression vector S11-CapG-IN or S11IN for controls using the FUGENE-6 transfection reagent according to the manufacturer's instructions (Roche, Mannheim, Germany). 48 hours after transfection, supernatants were filtered through a 0.45 *μ*m filter and 1 mL of virus solution was given to Hey cells for stable retroviral transfection. Cell selection was carried out over 7 days using Neomycin.

### 2.5. SiRNA Mediated CapG Knockdown

SK-OV-3 cells were disseminated in 6-well plates at a concentration of 5 × 10^4^ cells per well containing 2 mL serum free medium (24 hrs). For transfection, 12.5 nM siRNA (sequence 5′-ggu ggu gug gag uca gca u-3′, binding within CapG coding region 335-373, Ambion, UK) was added using the HiPerfect transfection reagent (Qiagen, Germany) according to manufacturer's protocol. Optimal siRNA concentration was tested before and revealed best results after 5 days. siRNA effects were confirmed by qRT-PCR and Western blot analyses.

### 2.6. Wound Healing Assay (Scratch Assay)

Cells were allowed to form confluent layers in standard 6-well plates. Then, cells were rinsed and a horizontal reference line was drawn in the middle of each well. Three vertical scratches were made per well using a 200 *μ*L pipette tip. For documentation purposes, each scratch was measured at baseline. Cells were cultured in medium (with halved serum concentration) for 24 and 72 hours, respectively, until first wound closure was apparent. Wells were washed again with PBS three times and cells were fixed in place by methanol, stained with toluidine blue (0.1%) and wound closure was measured. Experiments were carried out in triplicates and repeated three times.

### 2.7. Matrigel Transwell Invasion Assay

Cells were harvested and suspended in FBS-free RPMI1640 medium at a concentration of 1 × 10^5^ cells for transwell invasion assays using BD Biocoat Matrigel invasion chambers (BD Biosciences, USA) following the manufacturer's instructions. Briefly, 500 *μ*L of the cell suspension was added to the upper compartment, while the lower compartment contained 750 *μ*L medium with EGF (15 ng/mL) additionally. After 22 hours of incubation, chambers were rinsed and the upper surface of the membrane was scrubbed with moistened cotton swabs to remove Matrigel matrix and noninvading cells. Afterwards, cells on the lower surface were fixed using methanol and stained with 0.1% toluidine blue. Membranes were cut out and placed on microscope slides for microscopic evaluation and documentation (AxioVision Software, Zeiss, Germany).

### 2.8. SDS PAGE and Western Blot

Each protein sample (20 *μ*g protein) was processed using the Protein II apparatus (BioRad, Munich, Germany) and according to the Laemmli protocol [18] on 6–12% SDS-polyacrylamide gels. Proteins were then transferred on nitrocellulose membranes (Hybond, GE Healthcare, Freiburg, Germany) and unspecific bonding sites were blocked by Western Blocker Solution (Sigma-Aldrich, Germany). CapG antibody (rabbit IgG, polyclonal) was added and detected by anti-rabbit IgG-HRP (both Santa Cruz Biotech, USA), each in a 1 : 2500 dilution. *α*-Tubulin antibody (rabbit IgG; Sigma-Aldrich, Germany) and corresponding anti-rabbit-IgG-HRP were used at a 1 : 4000 dilution. Visualization was performed on films (Hyperfilm, GE Healthcare, Munich, Germany) in darkrooms.

### 2.9. HRM Analyses

After amplification of the target CapG sequence via qRT-PCR, HRM analyses were performed on Fast-RealTime-PCR Systems (Applied Biosystems; USA) detecting allele specific dsDNA dissociation curves. Previously, reference cell lines were analyzed by DNA sequencing and allele corresponding dissociation curves were identified (T47D A/A, Hec1A G/G, MDAMB231 G/A). For analyses, 2 *μ*L DNA sample, 0.6 *μ*L SYTO9 (Life Technologies, USA), and 0.2 *μ*L AmpliTaq Gold DNA polymerase (Life Technologies, USA) were added to primers and buffer.

### 2.10. Statistical Analysis

Statistical analyses and graphic visualization of present data were performed with the R software (R Project for Statistical Computing; http://www.r-project.org/; Vienna, Austria) using the students *t*-test.

## 3. Results

### 3.1. Overexpression of CapG in OC Samples

**Figure 1:**
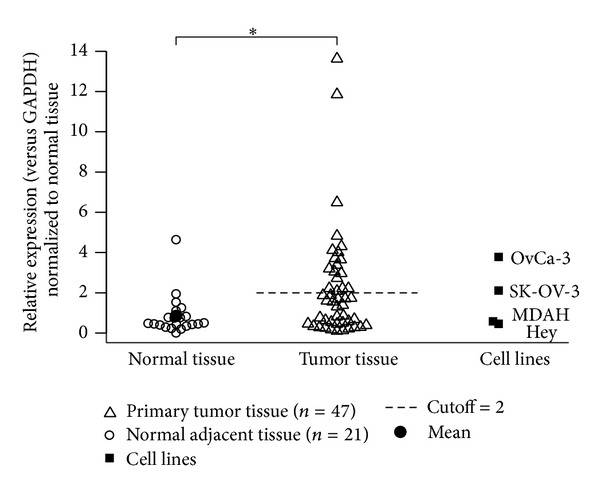
CapG expression in normal and OC tumor tissues. CapG expression in primary tumor tissue samples from 47 patients with OC was analyzed by qRT-PCR and compared to normal adjacent tissue samples (*n* = 21). Single probes were normalized to housekeeping gene GAPDH. Mean expression in normal tissue was calculated and standard deviation added for upper cutoff (dashed line). Thus, expression levels greater than 2.0-fold above mean CapG expression in controls was defined as overexpression and 38% of all tumor samples display such results. To the right, CapG expression of ovarian carcinoma cell lines OvCa-3, SK-OV-3, MDAH, and Hey. Each sample was analyzed in duplicates and repeated three times.

CapG expression was determined in OC samples (*n* = 47 serous OC) and in adjacent tissues (*n* = 21). CapG results of each sample were normalized to the housekeeping gene GAPDH. Mean CapG expression level was calculated in normal tissue samples and CapG expression in each tumor sample was compared to mean expression in controls, consecutively (Figure 1).

Mean CapG expression in controls was complemented by standard deviation. Thus, the cutoff for CapG overexpression in tumor samples was set at a 2.0-fold increase of CapG expression compared to the mean expression level in the control probe set.

In 18/47 (38%) of the OC samples, CapG expression was determined above this cutoff.

### 3.2. CapG Expression in Ovarian Carcinoma Cell Lines

To establish a functional CapG expression* in vitro* model OC cell lines Hey, SK-OV-3, MDAH, and OvCa-3 were analyzed by qRT-PCR with respect to their CapG expression normalized against GAPDH gene expression (Figure 1) and compared to the mean expression level in normal tissues. Hey cells showed the lowest CapG expression (0.25-fold); SK-OV-3 and OvCa-3 cells exceeded normal expression by more than 2-fold. Additionally, CapG expression in OC cell lines was in the same range compared to the OC tissue samples.

### 3.3. CapG Expression Modulates OC Cell Migration and Correlates with Invasiveness

**Figure 2:**
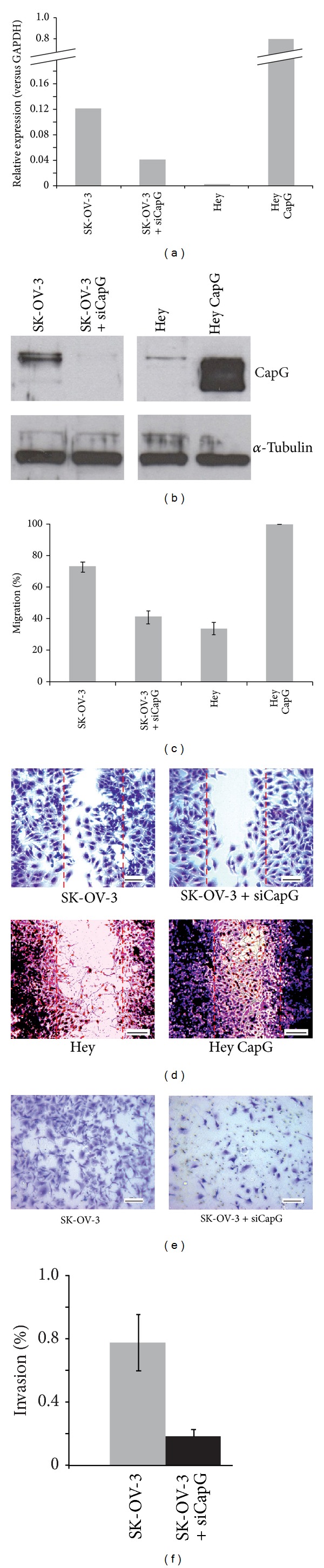
(a) Relative CapG expression of ovarian cancer cell lines SK-OV-3 and Hey, prior to and after alteration of CapG expression using CapG mediated siRNA (SK-OV-3 + siCapG) and stable retroviral transfection (Hey CapG), respectively. Values were calculated performing qRT-PCR in duplicates, repeated three times. Each probe was normalized to the housekeeping gene GAPDH. (b) Effects were confirmed by Western blot analyses. Equal detection of *α*-tubulin in samples verified equality of protein quantity. (c, d) Performing a migration assay (scratch assay), the motility in CapG siRNA treated cells was nearly halved in 24 hrs while overexpression in Hey cells led to a significantly earlier closure of the scratch after 72 hrs. (e, f) The invasiveness of siRNA treated cells was also significantly decreased in the transwell Matrigel invasion assay (by 4-fold) in SK-OV-3 cells.

To estimate the impact of CapG expression on migration in ovarian tumor cell lines, the intrinsically low expressing cell line Hey was stably transfected with a retroviral expression vector (S11-CapG-IN), which resulted in 230-fold higher expression of CapG in Hey cells (confirmed at mRNA level by qRT-PCR and at protein level by Western Blot analysis). In contrast, SK-OV-3 cells (with high endogenous CapG expression) were transfected with CapG specific siRNA. A 4-fold reduction in CapG expression was verified by qRT-PCR and Western Blot, respectively (Figures 2(a) and 2(b)).

In wound healing assays, CapG overexpression led to a significantly increased migration rate within 72 hrs compared to untreated Hey cells (by 2.94-fold). Furthermore, CapG knockdown in SK-OV-3 cells decelerated time to complete wound closure (reduction by 0.56-fold). An overall difference was detected between the motility among both cell lines with SK-OV-3 displaying a 2-3-fold increase in migration potential (Figures 2(c) and 2(d)).

For further investigation of comparably small changes in migration rate in SK-OV-3 cells following CapG knockdown, invasiveness was assessed in this cell line by Matrigel transwell invasion assays. After 24 hours, SK-OV-3 cells revealed a significant decrease in invasiveness (to 0.25-fold) indicated by fewer cells transmigrating the BME matrix (Figures 2(e) and 2(f)).

Thus, cellular migration as well as invasiveness was positively correlated with CapG expression levels.

### 3.4. Single Nucleotide Polymorphism rs6886 is a Putative Link between Ovarian and Fallopian Carcinoma

SNP rs6886 (c.1004G > A) is located in exon 10 of the CapG gene (chromosome 2) affecting the first codon within a protein kinase C phosphorylation recognition motif [R_335_ES_337_]. This results in an amino acid exchange p.Arg335His destroying the PKC-recognition motif and consequently in a loss of Ser_337_ phosphorylation (unpublished results). This is the first time that a functional effect of rs6886 could be identified. So far, no association studies have been published with respect to oncologic aspects.

Here, we genotyped 263 DNA samples (isolated from EDTA blood samples) of OC patients and 107 DNA samples of healthy controls (age matched) by HRM analyses (Table 1). Results were compared to allele frequencies indicated in the HapMap database (http://www.hapmap.org/), where the homozygous A/A genotype (His) accounts for 8%, the homozygous G/G genotype (Arg) for 43%, and the heterozygous G/A genotype for 48% of all in a normal population.

Although results in OC and control cases were not significantly different (*P* = 0.153), a marked increase in minor A-allele frequency was detected in OC patients. The frequency of the homozygous A/A genotype was well above the own control sample set (16% versus 10%) and twice as high as in the HapMap dataset (8%) or in BC patients (6%, own unpublished data). Looking at the genotype of patients with fallopian tube tumors, half of all cases display the homozygous A/A genotype. Therefore, in this cohort, the homozygous histidine encoding variant (A/A) is significantly associated with fallopian tube carcinomas (*P* < 0,0001).

## 4. Discussion

In OC, diagnosis is usually late due to a lack of precursors and biomarkers. Just recently, a new classification has been established with type II tumors being aggressive with early invasive growth. Despite p53 mutations, no specific molecular alterations have been identified [2].

In a variety of tumors, overexpression of CapG has been demonstrated and recently the association of CapG overexpression and metastasis has been shown in colorectal cancer [19]. Performing an* in silico* expression profiling approach for identification of differentially regulated genes in gynecological cancer, CapG has been identified as a putative oncogene overexpressed in breast and ovarian cancer [13].

Moreover, recent microarray analyses have shown associations between CapG expression and tumor prognosis with CapG overexpression in deceased patients with stage III serous OC in comparison to normal CapG expression in tumors of a survivor cohort [20]. Underlining its crucial role in tumor progression, CapG expression was found elevated at the tumor invasion front, the so-called interface zone, in breast cancer particularly [21]. A correlation of CapG overexpression with cell motility and invasive phenotype of CapG overexpressing breast and pancreatic cancer cell lines was also shown previously [8, 12].

By capping actin polymers, CapG is a key player in cytoskeletal remodeling and membrane ruffling [17]. While cellular motility can be influenced through cytoplasmic CapG, the function of nuclear CapG remains unclear.* In vitro* experiments with epithelial kidney cells revealed that transfectants with modified CapG carrying a nuclear export sequence (NES) were less invasive [16].

Our study aims at investigating the effect of differential CapG expression in OC cells hypothesizing that CapG overexpression is involved in OC cell motility and invasiveness. In a sample set of 47 ovarian carcinomas and 21 normal tissue samples, CapG overexpression was determined in 38% of all tumor samples (18/47), which was concordant to previous results based on cancer profiling arrays [13]. The cutoff value was set at a standard deviation higher than the mean expression level determined in normal adjacent tissue samples (Figure 2). Mean CapG expression levels in tumor specimen and normal tissues differed significantly (*P* < 0.027) indicating the putative oncogenic function of CapG in ovarian carcinomas.

To establish an* in vitro* model for further functional characterization of CapG in ovarian carcinomas, four ovarian carcinoma cell lines were analyzed. CapG expression levels were comparable and within the range of the OC samples with a significant CapG overexpression in OvCa-3 and SK-OV-3 cells. The latter has been described as more aggressive with a higher intrinsic metastatic potential [22] and thus was chosen for in-depth analyses.

To demonstrate the impact of CapG expression on cell migration we investigated the effects of CapG overexpression in Hey transfectants and CapG depletion in SK-OV-3 cells in comparison to the parental cell lines.

Transfected Hey cells displayed a significant increase in motility in wound healing assays compared to untreated Hey cells. Concordant to that, the migration rate in CapG knockdown SK-OV-3 cells was reduced by nearly 50% (Figure 2). Results suggested that CapG overexpression has a higher impact on migration than CapG reduction. But looking at a markedly stronger effect of overexpression on mRNA level in transfected Hey cells (230-fold) compared to relatively small changes following CapG knockdown (to 0.25-fold only), the altered migration rate appears to be proportional to the differential CapG expression in the investigated cell lines. With some CapG protein still being expressed, residual migration potential in treated SK-OV-3 cells may be expected.

However, to further evaluate the impact of CapG depletion on cell migration, we analyzed siRNA treated SK-OV-3 cells in a Matrigel transwell invasion assay. Cells invading a basement membrane extract (BME)* in vitro* are thought to transmigrate the basement membrane* in vivo*. Thus, we analyzed the role of CapG not only with regard to migration properties but also to proinvasive characteristics of an aggressive cell type [23]. Recent studies have shown that these CapG characteristics are most probably distinct from its major biological function [16] and possibly require nuclear CapG regulation.

The even more pronounced reduction (75%) of the invasive phenotype of SK-OV-3 cells after CapG depletion is in good agreement with the results of the scratch assay. It suggests that CapG might play an important role in migration as well as tumor invasion also in OC. Mechanistically, it remains to be explored how CapG influences the invasive behavior of cells. It is conceivable that tumor migration and proliferation rely on increased cancer cell motility through accelerated cytoplasmic actin turnover. On the basis of recent findings by that the nuclear fraction seems to be crucial for invasiveness, a key role of CapG in the nucleus is also possible [24]. By subcellular fractioning this matches with our findings (unpublished data) that SK-OV-3 cells, which display a highly invasive phenotype, contain a considerable amount of nuclear CapG protein. This becomes even more evident since nuclear complexes have been identified to carry nuclear actin and can be modified by actin-binding proteins such as CapG [25]. One of them is the BAF (Brg1/Brm-associated factor) complex which modulates DNA transcription in the nucleus [26, 27]. The molecular relationship of the subcellular localization of CapG and an invasive phenotype remains a challenging topic for in-depth investigations.

Since these results suggest that CapG modulates invasiveness in OC cell lines, we further strived to explore molecular mechanisms that contribute to CapG functioning.

Phosphorylation of CapG protein was already described as a potential posttranslational modification of functional relevance [28]. Using prediction tools like NetPhosK (http://www.cbs.dtu.dk/services/NetPhosK/) and PhosphoMotif finder (http://www.hprd.org/PhosphoMotif_finder), we identified potential phosphorylation sites of the CapG protein sequence. Amino acid Ser_337_ was identified as a potential phosphorylation site within a protein kinase C phosphorylation site recognition motif. This motif including Arg_335_ and consequently phosphorylation of Ser_337_ is affected by SNP rs6886 (p.Arg335His). For further evaluation of SNP rs6886, we carried out an association study, analyzing allele frequencies in gynecological tumor entities in order to reveal indications for an oncologic impact of this polymorphism. So far, the SNP has been associated with increased intima media thickness in carotid ultrasounds [29] but has not been investigated on other backgrounds.

Comparing 263 OC cases and 107 controls by genotyping SNP rs6886 performing HRM analyses, the MAF (A allele) was higher in OC cases (37.5%) and FTC cases (54%) compared to controls (31%). Even more evident was the genotype distribution with the homozygous A/A genotype expressing the histidine variant in 50% of FTC cases compared to 10.3% in controls (*P* < 0.0001). Interestingly, no differences in allelic frequencies were found in BC samples. Despite the low number of cases/controls investigated in this study, these results suggest a functional relevance of SNP rs6886 in OC and FTC. Because of the rarity of FTC, only 12 samples were available and confirmation of these results in larger probe sets in future studies is needed.

Although its relevance is yet not understood, this polymorphism might hint to a possible link between OC and FTC. In case CapG phosphorylation at Ser_337_ affects cell motility and invasiveness, the CapG Arg_335_ variant with intact PKC phosphorylation recognition site may be relevant for migration of tumor cells from fallopian tubes into the ovaries. Recent findings have opened the discussion whether the fallopian tube might be point of origin of the serous OC. The development of ovarian cancer in Dicer-Pten (crucial for miRNA synthesis) double-knockout mice could be inhibited by removal of the fallopian tube* in vivo* [30]. Supporting these findings, the gene expression profiles of experimentally induced carcinomas in mice and serous high-grade OC in women resemble to a high degree. In a set of 41 serous high-grade tumors, 59% also displayed serous tubal intraepithelial carcinomas (STICs) [31].

## 5. Conclusion

In this study, CapG overexpression in 38% of OC was found and its functional relevance on cell migration as well as invasiveness has been clearly demonstrated. Although mechanisms are still to explore, the positive correlation of SNP rs6886 as a possible functional relevant CapG variant with FTC may introduce a novel interesting link between ovarian and fallopian tube carcinomas. However, further studies are needed to validate the association of SNP rs6886 with OC and FTC and to evaluate CapG as a prognostic biomarker.

## Figures and Tables

**Table 1:** Genotype frequencies of the single nucleotide polymorphism SNP rs6886.

SNP type	CTRL		OC		FTC		BC		HapMap
	*n*=	%	*n*=	%	*n*=	%	*n*=	%	%
homozygous-His (A/A)	11	10.28	42	15.96	6	50.00	7	6.14	8
homozygous-Arg (G/G)	51	47.66	107	40.68	5	41.66	55	48.24	43
heterozygous (G/A)	45	42.05	114	43.34	1	8.33	52	45.61	48
Total	**107**	**100**	**263**	**100**	**12**	**100**	**114**	**100**	**100**

SNP rs6886 (c.1004A>G, p.His335Arg) genotype frequencies were determined in healthy controls (CTRL), ovarian carcinoma (OC), fallopian tube carcinoma (FTC), and breast cancer (BC) cases compared to the published data of the International HapMap Project (http://www.hapmap.org/). CapG reference sequence (NM_001747.3) was obtained from the RefSeq database (http://www.ncbi.nlm.nih.gov/refseq/).

## Abbreviations

Arg: Arginine
BC: Breast cancer
BME: Basement membrane extract
CTRL: Control
EGF: Epithelial growth factor
FTC: Fallopian tube carcinoma
GAPDH: Glyceraldehyde 3-phosphate dehydrogenase
His: Histidine
HRM: High resolution melting
KDa: Kilodalton
MAF: Minor allele frequency
OC: Ovarian carcinoma
PBS: Phosphate buffered saline
PIP_2_: Phosphatidylinositol 4,5-bisphosphate
PKC: Protein kinase C
Pten: Phosphatase and tensin homolog
qRT-PCR: Quantitative realtime-PCR
RefSeq: Reference sequence
SiRNA: Small interfering RNA
SNP: Single nucleotide polymorphism
STIC: Serous tubal intraepithelial carcinoma.
